# Exercise Volume and Coronary Artery Calcification: A Systematic Review

**DOI:** 10.1016/j.cjco.2025.12.009

**Published:** 2025-12-30

**Authors:** Chase J. Ellingson, Jyotpal Singh, M. Abdullah Shafiq, Neha Mehta, Sabiha Sultana, J. Patrick Neary, Yang Zhan, Amir Ahmadi, Matthew J. Budoff, Payam Dehghani

**Affiliations:** a College of Medicine, University of Saskatchewan Regina Campus, Regina, Saskatchewan, Canada; b Prairie Vascular Research Inc., Regina, Saskatchewan, Canada; c Faculty of Kinesiology and Health Studies, University of Regina, Regina, Saskatchewan, Canada; d Mount Sinai Heart at St Luke's Hospital, New York, New York, USA; e The Lundquist Institute at Harbor-UCLA Medical Center, Division of Cardiology, Los Angeles, California, USA

**Keywords:** coronary artery calcification, coronary artery disease, exercise volume, endurance training, physical activity, atherosclerosis, masters athlete

## Abstract

**Background:**

Exercise confers numerous health benefits, including a reduction in all-cause and cardiovascular (CV) mortality. However, conflicting evidence suggests that high-volume endurance exercise may increase coronary artery calcification, a robust predictor of CV events. This systematic review investigated the relationship between exercise volume and coronary artery calcium (CAC) scores.

**Methods:**

A systematic search of Medline, Embase, PubMed, and the Cochrane Library (1990 to November 26, 2025) was conducted. Studies reporting exercise volume and CAC scores were included. The methodological index for nonrandomized studies (MINORS) scale was implemented to test study quality. Exercise volume (minutes per week) was stratified into 4 categories: low; moderate; moderate-high; and high.

**Results:**

A total of 33 studies met the inclusion criteria: 15 reported higher CAC scores in their highest-volume groups; 8 showed no association or inverse associations; and 10 included single-cohort data. Nine of 12 comparative studies with participants exercising > 450 min/wk showed higher CAC scores in high-volume exercisers. The majority of the 9 studies reporting clinical outcomes showed no relationship or an inverse association between exercise volume and mortality or CV events. Among 5 studies assessing plaque composition, 4 reported a more benign, calcified plaque composition among their high-volume exercisers, representing a potential mechanism for the lower risk of CV events and mortality reported in this population, compared to that of less-active individuals with similar CAC scores.

**Conclusions:**

High-volume exercisers may have higher CAC scores compared to less-active cohorts. Despite elevated CAC scores, lower mortality and CV event rates observed in these groups challenge the clinical significance of this observation.

**Registration:**

PROSPERO CRD42024607693.

## Central Illustration

**Figure:**
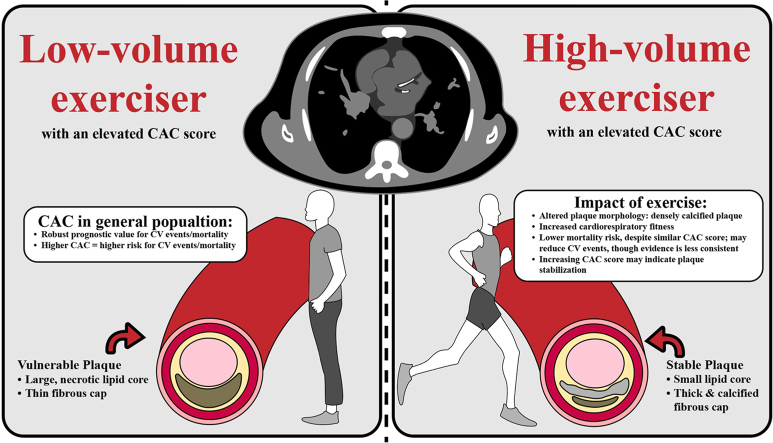

## Résumé

**Contexte:**

L'exercice physique confère de nombreux bénéfices pour la santé, notamment une réduction de la mortalité toutes causes confondues et de la mortalité cardiovasculaire (CV). Cependant, des données contradictoires suggèrent que la pratique intensive d'exercices d'endurance pourrait augmenter la calcification des artères coronaires, un facteur prédictif important d'événements CV. Cette revue systématique a examiné la relation entre le volume d'exercice et le score calcique coronaire (SCC).

**Méthodologie:**

Une recherche systématique a été effectuée dans Medline, EMBASE, PubMed et Cochrane Library (de 1990 au 26 novembre 2025). Les études rapportant le volume d'exercice et les SCC ont été incluses. L'échelle MINORS (methodological index for non-randomized studies) a été utilisée pour évaluer la qualité des études. Le volume d'exercice (min/semaine) a été stratifié en quatre catégories : faible, modéré, modéré-élevé et élevé.

**Résultats:**

Trente-trois études répondaient aux critères d'inclusion : 15 études rapportaient des SCC plus élevés dans leurs groupes au volume le plus élevé; huit études ne montraient aucune association ou une association inverse; 10 études incluaient des données provenant d'une seule cohorte. Neuf des 12 études comparatives dont les participants faisaient plus de 450 minutes d'exercice par semaine ont montré des SCC plus élevés chez les personnes à volume élevé d'exercice. La majorité des neuf études rapportant des résultats cliniques n'ont montré aucune relation ou une association inverse entre le volume d'exercice et la mortalité ou les événements CV. Parmi les cinq études évaluant la composition des plaques, quatre ont rapporté une composition plus bénigne et calcifiée des plaques chez les personnes pratiquant un volume d'exercice élevé, ce qui pourrait expliquer le risque plus faible d'événements CV et de mortalité rapporté dans cette population par rapport aux personnes moins actives ayant des SCC similaires.

**Conclusion:**

Les personnes pratiquant un volume d'exercice physique élevé peuvent présenter des SCC plus élevés que les cohortes moins actives. Malgré des SCC élevés, les taux de mortalité et d'événements CV plus faibles observés dans ces groupes remettent en question la signification clinique de cette observation.

Exercise offers numerous health benefits and is associated with reduced all-cause and cardiovascular (CV) mortality.^1^, ^2^, ^3^ However, recent research indicates that high-volume exercise, paradoxically, may be associated with increased coronary artery calcium (CAC) scores.^4^, ^5^, ^6^, ^7^, ^8^, ^9^, ^10^ Calcification within the coronary arteries often reflects advanced atherosclerotic lesions,^11^ and CAC scores are used as a surrogate marker for coronary artery disease (CAD).^12^ In addition, CAC scores are a robust predictor of mortality and CV events in the general population.^13^^,^^14^ The pathophysiology underlying coronary calcification involves inflammation of the vasculature, oxidative stress, and lipid accumulation, ultimately promoting osteogenic differentiation of vascular smooth muscle cells.^11^

Although the biological mechanisms linking exercise and CAC remain incompletely understood, several hypotheses have been proposed. Repeated mechanical stress to the coronary vasculature, driven by increased heart rate and cardiac contractility during exercise, may cause endothelial injury and accelerate atherosclerosis.^11^^,^^15^^,^^16^ Exercise also induces transient inflammation and oxidative stress, which may be beneficial acutely but could promote vascular calcification if chronically elevated.^11^^,^^16^ The level of parathyroid hormone, a principal regulator of calcium homeostasis, rises transiently with exercise and could contribute to calcification in this population.^11^^,^^15^^,^^16^ Additional proposed mediators include alterations in steroid hormones, environmental exposures, diet, genetics, and psychological stress.^11^^,^^15^, ^16^, ^17^, ^18^

The notion that exercise may increase CAC scores has created confusion among the general population regarding the safety of exercise. To address the ambiguity in this area, we conducted a systematic review focused on exercise volume and CAC scores. By exploring this relationship, we hope to provide further clarity for clinician-patient shared decision-making and counselling on the benefits and potential risks of prolonged high-volume exercise.

## Methods

All supporting data are available within the article and its accompanying supplemental material. This review was registered in the International Prospective Register of Systematic Reviews (PROSPERO, #CRD42024607693). The protocol was developed using the Preferred Reporting Items for Systematic Reviews and Meta-Analyses (PRISMA) guidelines (Supplemental Appendix S1). Two researchers (C.J.E. & M.A.S.) independently extracted, recorded, and assessed data, and a third researcher (P.D.) adjudicated disagreements.

### Search strategy and study selection

The original systematic search was completed on October 19, 2024, and an updated search was done on November 26, 2025, using Medline, Embase, PubMed, and the Cochrane Library. The date range was set as January 1990 to November 26, 2025, as the earliest work on CAC scoring was published in 1990.^19^ The search strategy was to use the following string: ((exercise) OR (endurance exercise) OR (physical exertion) OR (aerobic exercise)) AND ((coronary artery calcification) OR (calcium score) OR (coronary plaque)). Non-English articles were excluded. References of eligible articles and previous related reviews were searched.

Eligible articles had to report exercise volume (intervention) and CAC scores (primary outcome). Exclusion criteria were incomplete information, case reports, and preclinical studies. Within included studies, cohorts with no reported exercise participation were excluded.

### Data extraction and analysis

Search results were imported into an EndNote (Clarivate Analytics, Philadelphia, PA) referencing file, and duplicate data were removed. Two independent researchers (C.J.E. and M.A.S.) extracted data using a preconstructed data collection tool. Data recorded on the collection tool included article citation, reason for rejection, demographics, sample size, study type and/or design, intervention details (mode(s), volume, and years of exercise), CAC score, summary of the main findings, sex differences, mortality, and CV events.

**Figure 1:**
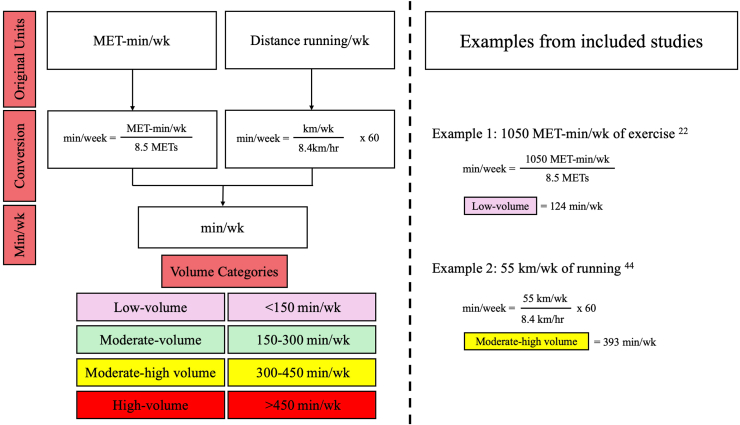
Conversion of exercise volume metrics and classification into standardized categories. This figure illustrates the method used to standardize reported exercise volume across studies, converting original units (metabolic equivalent of task [MET]-minutes per week and distance run per week) into minutes per week. MET-minutes were converted assuming vigorous intensity exercise (8.5 METs), and running distances were converted using an average running speed of 8.4 km/h, based on the *Compendium of Physical Activities*.^19^ Standardized values were then categorized into 4 volume strata: low (< 150 min/wk), moderate (150-300 min/wk), moderate-high (300-450 min/wk), and high (> 450 min/wk). Examples from included studies demonstrate this process.

We investigated the relationship between exercise volume (minutes per week) and CAC scores. Based on the *Compendium of Physical Activities,*^20^ several assumptions were made to convert studies to a standardized volume measure. To convert studies reporting exercise in metabolic equivalents of task (MET)minutes per week to minutes per week, exercise intensity was assumed to be vigorous (8.5 METs),^20^ which is appropriate for the predominantly active populations studied and allows for standardized comparison. In studies reporting volume in distance per week of running, we assumed an average pace of 8.4 km per hour (equivalent to 8.5 METs;^20^ Fig. 1). Using the Canadian Society for Exercise Physiology recommendation of 150 minutes of moderate-to-vigorous physical activity per week,^21^ we created the following categories to classify exercise volume: low (< 150 min/wk), moderate (150-300 min/wk), moderate-high (twice the recommended volume; 300-450 min/wk); and high (3 times the recommended volume; > 450 min/wk).

Narrative summaries were compiled to present the results of the systematic review. The I^2^ statistic and the Egger’s Test were used to assess heterogeneity. Heterogeneity was high (I^2^ = 99.96%), and the funnel plot (Supplemental Fig. S1) and Egger’s Test (*P* = 0.02) suggested significant asymmetry and heterogeneity. Therefore, we did not conduct a meta-regression.

### Risk-of-bias assessment for systematic reviews

The methodological index for nonrandomized studies (MINORS) scale was utilized to assess study quality. Two independent reviewers (C.J.E. and J.S.) assessed study quality, and a third independent reviewer (P.D.) adjudicated disagreements.

## Results

### Characteristics of included studies

**Figure 2:**
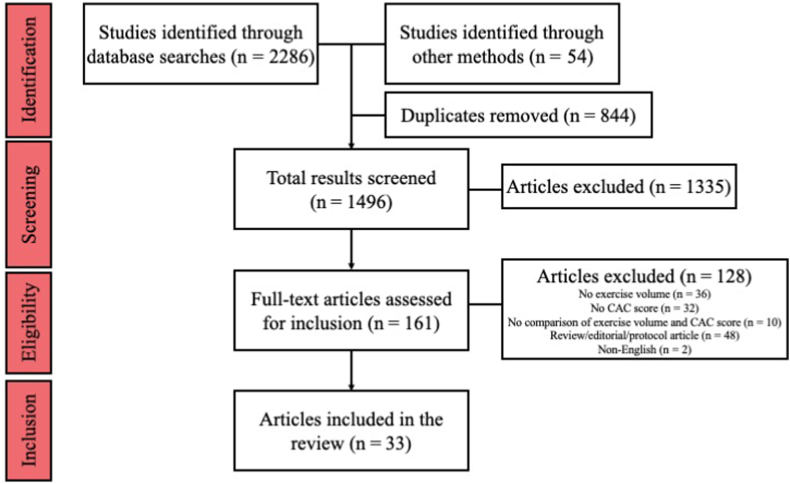
Preferred Reporting Items for Systematic Reviews and Meta-Analysis (PRISMA) flow diagram. CAC, coronary artery calcium.

Of the 33 included studies, 25 and 24 reported CAC scores in absolute and relative values, respectively. Twenty-two studies were cross-sectional, and 11 were prospective, with a total of 170,176 participants (63% male), with a mean age range of 40.4-69.8 years. Twenty-two studies adjusted for confounding variables in their associations of CAC scores to exercise volume (Supplemental Table S1). The confounders most commonly adjusted for included age (58% of studies), body mass index/obesity (58% of studies), dyslipidemia/lipid values (58% of studies), hypertension/blood pressure (55% of studies), smoking (52% of studies), sex (33% of studies), diabetes mellitus (33% of studies), and lipid-lowering therapy/statin use (27% of studies). Among 14 studies, the physical activity was primarily running and cycling. The following sections detail the studies reporting exercise volume and its relationship to CAC scores, along with secondary outcomes of mortality/CV events, and sex differences. The PRISMA flow diagram is shown in Figure 2.

### Exercise volume and increased coronary artery calcium scores

Overall, 15 studies, with a cumulative sample size of 153,790, found higher CAC scores in higher-volume exercisers. In one study of low-volume exercisers with 4 separate cohorts, CAC was noted in 45.2% (60 min/wk), 50.2% (70 min/wk), 43.5% (77 min/wk), and 57.4% (100 min/wk), with the highest proportion of CAC observed in those exercising the most.^22^ One study found an increased prevalence of CAC among moderate-volume exercisers, reporting CAC scores > 0 in 41.1% of moderate-volume exercisers, compared to 39.4% in their lowest-volume cohort.^23^

Four studies examining moderate-high volume exercisers noted higher proportions of elevated CAC scores compared to those in less-active cohorts.^5^^,^^10^^,^^24^^,^^25^ One study in adults engaged in regular sport identified an association between CAC scores and exercise volume, reporting an adjusted odds ratio (OR) of 1.02 (95% confidence interval [CI] 1.01-1.04; *P* = 0.006) per MET-hour per week, and 3.20 (95% CI 1.56-6.57; *P* = 0.001) in a comparison of > 2000 MET-min/wk to < 1000 MET-min/wk.^5^ Another study found that 22.9% of their moderate-high-volume exercisers had a CAC score > 100, compared to 18.1% in their lowest-volume cohort.^10^ In addition, the mean CAC score among moderate-high-volume exercisers was 24.3 (standard deviation, 116.9) compared to 14.3 (standard deviation, 71.0) in low-volume exercisers (*P* < 0.001).^24^ Finally, a significantly higher proportion (*P* < 0.001) of moderate-high-volume exercisers (21.78%), as compared to low-volume exercisers (16.16%), had a CAC score of > 0.^25^

Twelve studies examined high-volume exercisers (> 450 min/wk), with 9 demonstrating higher CAC scores in their highest-volume cohorts.^6^, ^7^, ^8^, ^9^^,^^26^, ^27^, ^28^, ^29^, ^30^ Higher CAC scores were observed in ultramarathon and marathon runners performing high-volume exercise, compared to those for non-marathon runners (OR = 10 (95% CI 2.30-43.45; *P* = 0.002) for CAC scores in the ≥ 50th percentile).^28^ Also, higher CAC scores were observed among lifelong male endurance athletes exercising at a high volume, compared to those of healthy controls (8.5 and 1.3, respectively).^6^ One study reported the age-adjusted OR of CAC > 0 was 1.76 (95% CI 1.35-2.29) in their high-volume exercisers.^7^ Another study in masters-level athletes noted the median CAC score of high- and low-volume exercisers was 0; however, among those with CAC > 0, median CAC scores were higher in male high-volume exercisers compared to low-volume controls (86 and 3, respectively; *P* = 0.02).^8^ In healthy male participants, high-volume exercise was associated with 19.9% (95% CI 10.1-29.7%; *P* < 0.001) higher mean CAC scores.^9^ Additionally, 85.8% of high-volume exercisers had CAC scores of 0, compared to 91.4% of low-volume exercisers.^27^ A comparison of marathon runners exercising at high volumes to age-matched controls exercising at a moderate volume showed no differences in median CAC scores (36 and 38, respectively; *P* = 0.36); however, the CAC scores in the marathon runners were higher compared to those in age and risk-factor-matched controls (36 and 12, respectively; *P* = 0.02).^29^ Also, a group of male high-volume exercisers had an 11% higher likelihood of having a CAC > 100 (relative risk = 1.11; 95% CI 1.03-1.20), compared to their low-volume counterparts.^26^ Finally, in a study of adults without known CAD, high-volume exercisers had the highest CAC scores, with a mean CAC of 266.5 (*P* = 0.03); the prevalence of CAC > 100 was similarly higher in the high-volume group (34.5%), compared with that in the low-volume group (28.5%).^30^

### Exercise volume and no change or decrease in CAC scores

Eight studies (n = 14,695) found no association or decreased CAC scores in their highest-volume exercisers. In low-volume exercisers, a strong inverse association was found between exercise duration and CAC scores; in this study, the median CAC score in the higher-volume cohort was 11, whereas the lower-volume cohort had a median CAC score of 18 (*P* < 0.002).^31^ Additionally, 3 studies of moderate-volume exercisers found no association between CAC scores and exercise volume.^32^, ^33^, ^34^ One study reported a CAC score of 27.7 in their moderate-volume cohort, compared to 57.1 in low-volume exercisers and 45.5 in sedentary individuals (*P* = 0.23).^32^ Similarly, another study highlighted CAC scores of 29.9 in moderate-volume exercisers, compared to 109.4 in sedentary controls (*P* = 0.055).^33^ A study also found an inverse relationship between accelerometer-measured physical activity and the development of CAC; however, this association was not replicated when exercise volume was assessed using self-reported measures.^34^ One study of moderate-high-volume exercisers found no association between exercise volume and increasing CAC scores.^35^ In a comparison of 2 groups of low-volume exercisers to 1 group of moderate-to-high-volume exercisers, no association was present between exercise volume and CAC scores.^35^

Three studies, including high-volume exercisers, found no association between exercise volume and CAC scores.^36^, ^37^, ^38^ Among high-volume exercisers with CAC < 100 and ≥ 100, no associations with increased CAC scores, compared to lower-volume cohorts, were identified, with *P*-values of 0.25 and 0.86, respectively.^36^ Additionally, in a study that divided cohorts into quintiles of exercise volume, no association was noted between high-volume exercise and elevated CAC scores, with mean CAC scores of 95 in high-volume exercisers and 71 in the low-volume cohort (*P* = 0.65).^37^ Finally, compared to healthy controls exercising at a low volume, female masters athletes performing high-volume exercise were less likely to have a CAC score in a > 50th percentile (19% vs 32%, *P* = 0.03) and a > 75th percentile (14% vs 25%, *P* = 0.045).^38^

### Single-cohort studies

The remaining 10 studies (n = 1691) did not provide comparisons between cohorts completing different volumes of exercise. Among low-volume exercisers with CAC > 0, CAC scores were 1-100, 101-400, and > 400, in 48.1%, 25.8%, and 26.1% of the population, respectively.^39^ In active middle-aged asymptomatic male individuals engaged in moderate-volume exercise, approximately 1 in 5 had occult CAD, with CAC scores of 0, 16, and 39 stratified by age categories of 45-55, 55-64, and 65-79 years, respectively.^40^ A study of moderate-volume exercisers found median CAC scores of 1 at baseline and 31 after 6 years of follow-up care.^4^ In healthy adults aged ≥ 45 years engaged in moderate-volume exercise, a median CAC score of 31 was reported, and a positive linear correlation was observed between increasing exercise volume and higher CAC scores.^41^ In another group of moderate-volume exercisers, the median CAC score was 185, with 35% of the cohort having a CAC score ≥ 300.^42^ One study of 50 marathon runners exercising at a moderate volume reported a mean CAC score of 43.5, despite an otherwise favourable CV risk profile.^43^ In another cohort of marathon runners performing moderate-high-volume exercise, an average CAC score of 36 was reported, with a CAC score > 100 in 37% of the group.^44^ Among Norwegian mountain biker racers performing moderate-high-volume exercise, 70.5% had CAC scores of 0, and the median CAC score in the other 29.5% was 40.^45^ In veteran endurance athletes who perform high-volume exercise, 59% had CAC scores of 0, and the average CAC score in the remainder of this group was 34.^46^ In a cohort of female marathon runners exercising at high volumes, 21 of 26 had CAC scores of 0, with the remaining 5 participants having a mean CAC score of 1.4.^47^

### Secondary outcomes

#### Mortality and cardiovascular events

Of the 33 studies in the systematic review, 9 reported mortality and/or CV events (n = 93,325). In a cohort of 108 marathon runners, no deaths were observed over 21 months of follow-up evaluation.^29^ However, 4 runners, who had multiple cardiac risk factors, had cardiac events—2 experienced ventricular tachycardia during exercise, 3 required percutaneous coronary intervention, and 2 underwent coronary artery bypass grafting.^29^ In this cohort of marathon runners, event-free survival was inversely associated with CAC score (*P* = 0.018).^29^ Another study, which followed participants for 10 years, observed decreased mortality (hazard ratio [HR] = 0.52, 95% CI 0.29-0.91) in high-volume exercisers with CAC scores < 100; this same study found no increased risk of mortality in male high-volume exercisers with CAC scores of ≥ 100 (HR = 0.77, 95% CI 0.52-1.15).^26^ Additionally, this study found a decrease in all-cause mortality among high-volume exercisers with CAC scores > 1 (HR = 0.63, 95% CI 0.44-0.92).^26^ Further, a study observed no major adverse cardiovascular events (MACE) after 1 year of follow-up care in moderate- and low-volume exercise cohorts.^32^ One study found that exercise volume was inversely associated with MACE (HR = 0.88, 95% CI 0.79-0.98).^37^ Similarly, another study found a decreased risk of cardiovascular disease (CVD; HR = 0.72, 95% CI 0.56-0.94) and all-cause mortality (HR = 0.69, 95% CI 0.57-0.84) with high-volume exercise.^36^ Additionally, no association was found between MACE and exercise volume (HR = 1.01, 95% CI 0.44-2.31) in a study with 15 years of follow-up evaluation.^27^ Another study found no association between exercise volume and CV mortality in participants with CAC scores above 0 (HR = 0.81, 95% CI 0.36-1.84); however, higher exercise volume appeared protective in those with CAC scores of 0 (HR = 0.02, 95% CI 0.04-0.95).^25^ In a study of adults without known CAD, mortality was the lowest in the high-volume exercise group (HR = 0.71, 95% CI 0.60-0.83); however, the lowest risk of CV events was observed in their intermediate-volume exercise groups, suggesting that high-volume exercise may not be as protective for CV events as once thought.^30^ Lastly, lower mortality and CV event rates were observed in moderate-volume exercisers without preexisting CAC, whereas exercise did not appear to mitigate this risk if CAC was already present.^34^

#### Sex differences

Of the included studies, 21 included both male and female participants. Of these studies, only 5 reported sex differences (n = 79,267). In masters-level athletes, high-volume endurance exercise was associated with elevated CAC in male but not female individuals.^8^ In this study, the authors mention that only 30% of their population was female, and the age range suggested that the majority were premenopausal, which may have attenuated the risk of CAC with high-volume exercise.^8^ Additionally, 2 studies found that, unlike male high-volume exercisers, female high-volume exercisers are not more likely to have elevated CAC scores.^9^^,^^26^ In contrast, one study found that, like male high-volume exercisers, female high-volume exercisers also had elevated CAC scores compared to those of less-active controls.^28^ Lastly, after conducting sex-stratified analyses, one study found no association between exercise volume and CAD events in female exercisers, with the low event rate producing wide CIs that limit interpretability.^30^

### Bias assessment via MINORS

Of the 33 studies, 8 noncomparative studies had a mean MINORS score of 9.25, with the maximum attainable score being 16. The remaining 25 studies were comparative and had a mean MINORS score of 16.52, with the maximum attainable score being 24. The most common bias risks were the lack of a sample-size calculation, unbiased assessment of study outcomes, limited follow-up information, and limited baseline equivalence between groups. See Supplemental Table S2 for individual study MINORS scores. These results emphasize the importance of a cautious approach to interpreting this literature.

## Discussion

This systematic review is the most comprehensive assessment that has been conducted of the relationship between exercise volume and CAC scores. High-volume exercisers appear to have higher CAC scores compared to less-active cohorts^6^, ^7^, ^8^, ^9^^,^^26^, ^27^, ^28^, ^29^; however, a lower mortality incidence, and albeit less conclusively, a reduced risk of CV events^25^, ^26^, ^27^^,^^32^^,^^36^^,^^37^ raise questions regarding the clinical significance of elevated CAC scores in high-volume exercisers.

Fifteen comparative studies (n = 153,790; 91.3% of the comparative sample) reported a positive association between exercise volume and CAC scores, and only 8 studies (n = 14,695; 8.7%) reported no association or an inverse association. This reporting raises the question of whether underlying clinical or methodological differences may account for these divergent findings. Seventeen of the comparative studies utilized large epidemiologic cohorts of the general population; however, only 6 specifically recruited older athletes, of which 5 studies demonstrated a positive association between exercise volume and CAC scores. The one study that did not show a positive association was conducted in an all-female cohort, in whom this relationship may differ,^48^ although additional research is required to better elucidate potential sex-related differences. Additionally, the “negative” studies tended to include older participants (mean age 57.5 vs 53.5 years), with 3 studies including participants with multiple cardiac risk factors referred for cardiac risk assessment; in contrast, the “positive” studies enrolled patients free of clinical CVD. Given the strong influence of age on CAC,^49^ 16 comparative studies that adjusted for age were identified, of which 11 reported higher CAC scores with increasing exercise volume.^6^^,^^7^^,^^9^^,^^10^^,^^22^, ^23^, ^24^, ^25^, ^26^, ^27^, ^28^^,^^31^^,^^35^, ^36^, ^37^ Moreover, exercise volume was generally higher in the “positive” studies, with the average highest-volume cohorts completing 405 min/wk of exercise compared to 284 min/wk in the “negative” studies. Taken together, these differences suggest that the “negative” studies included older and higher-risk participants with lower exercise exposure, in whom preexisting CAD may have obscured the relationship between exercise volume and CAC scores. In contrast, healthier and higher-volume exercise cohorts tended to report positive associations more consistently. This fact underscores the importance of study population characteristics and exercise exposure when interpreting this literature.

The 9 studies in our systematic review examining CV events and/or mortality support the notion that although high-volume exercise may increase CAC scores, it confers a lower risk of mortality, and albeit less conclusively, a lower risk of CV events. Previous evidence suggested that high levels of cardiorespiratory fitness attenuate the risk of CVD at all CAC levels.^50^ However, 2 recent studies included in this analysis found that although a higher volume of exercise and cardiorespiratory fitness were associated with lower mortality, they did not decrease the risk of CVD in the presence of CAC.^30^^,^^34^ These recent findings call into question the notion that high-volume exercise reduces CV event risk despite elevated CAC; nonetheless, the observed mortality reduction with increasing exercise volume remains consistent. Further research is needed to better understand the relationship between exercise volume, CAC, and mortality and/or CV events. Sex-specific data remain limited; however, the majority of existing evidence suggests that, unlike men, female individuals may not exhibit increased CAC with higher exercise volumes.^8^^,^^9^^,^^26^^,^^38^ These sex differences are further supported by a recent meta-analysis on this topic.^48^

Several mechanisms may explain the coexistence of elevated CAC scores and lower CV events and/or mortality in high-volume exercisers. Multiple studies reported a more benign, calcified plaque composition among their higher-volume exercise cohorts with elevated CAC scores,^5^^,^^8^^,^^33^^,^^46^ which may help explain the lower mortality risk observed in this population and the possible, although less consistently reported, reduction in CV events, compared to that among less-active individuals with similar CAC scores.^36^^,^^37^ Similarly, higher levels of recreational physical activity were associated with higher average CAC density but not CAC volume, potentially reflecting a predominantly calcified plaque composition among exercising groups.^37^ However, one study found a higher burden of noncalcified and proximal plaques in lifelong athletes, calling this hypothesis into question.^6^ Some evidence suggests that high-volume exercisers may have larger epicardial coronary artery diameters, increased capillary density, and enhanced vasodilatory capacity,^51^, ^52^, ^53^ which may further reduce one’s risk in the presence of CAC. Two studies reported an inverse relationship between exercise volume and other measures of atherosclerotic CVD, such as plaque burden, stenosis severity, and high-risk plaques.^32^^,^^33^ Mixed evidence regarding plaque progression, a predictor of all-cause mortality,^54^ and the effect of increasing exercise volume has been reported; 3 studies have reported no association,^4^^,^^10^^,^^45^ whereas 2 studies have claimed a positive association.^24^^,^^27^ One study noted a positive association between exercise volume and progression of plaque density,^39^ which is inversely associated with the risk of CV events.^13^^,^^55^

Among statin users, plaque stabilization and reduced risk of CV events have been observed despite increasing CAC scores.^56^ This observation is attributed to the conversion of noncalcified plaques into more heavily calcified, stable plaques.^56^^,^^57^ In such cases, increasing CAC scores may indicate plaque stabilization rather than de novo formation of atherosclerotic plaques and/or plaque progression. Although speculative, a similar process, altering plaque composition, might occur in high-volume exercisers with elevated CAC scores (Central Illustration). In addition, accumulation of CAC in high-volume exercisers may, in part, represent chronic repair from subclinical coronary injury, due to increased mechanical stress on the vessel wall during exercise.^15^^,^^16^^,^^18^^,^^58^ As emphasized in a recent review, elevated CAC in highly active individuals requires cautious interpretation, as plaque stability may differ from that in sedentary populations.^59^ Incorporating measures of plaque volume and density, beyond Agatston scoring alone, may help improve risk assessment in this population.^59^

**Central Illustration:**
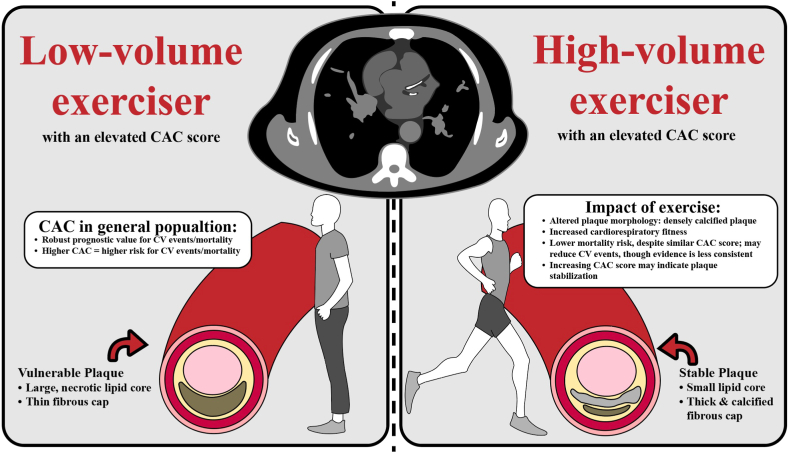
Differences in plaque morphology among high-volume exercisers. CAC, coronary artery calcium; CV, cardiovascular.

Strengths of this study include the large number of articles in the systematic review. In addition, our standardized approach to classifying exercise volume enabled us to stratify the results into categories based on established guidance from the Canadian Society for Exercise Physiology exercise recommendations.^21^ Although standardizing exercise volume using assumed MET values and running pace may introduce exposure misclassification, this approach allows direct comparison across a broad range of studies. Using a fixed MET value of 8.5 may underestimate exercise volume in individuals performing less-vigorous activity on average (general population) and overestimate it in those regularly engaged in higher-intensity exercise (endurance athletes). Although our systematic review focuses on exercise volume, we acknowledge that exercise intensity, although less thoroughly researched, may also play a role in CAC progression^4^; this represents a limitation of our approach. As evidenced by the relatively low MINORS scores and high heterogeneity (due to large variation in study results, standard deviations, and sample sizes), many methodological limitations exist in the current literature, warranting a cautious interpretation. Multiple studies utilized data from the same cohorts, albeit with differing subgroups, resulting in some overlap between samples included in this review. Considerable variability was present in the confounding variables adjusted for between studies, with strong CVD risk factors, such as smoking and diabetes, being controlled for in only 52% and 33% of studies, respectively. In addition, the relatively short follow-up duration for mortality and/or CV events may have limited this area of inquiry, as low event rates among these studies may limit interpretation. Additionally, given that mortality and CV events were not the primary outcome, all relevant literature pertaining to these outcomes may not have been captured in our search.

## Conclusions

Our systematic review suggests that high-volume exercisers may have higher CAC scores than less-active cohorts. However, the poor study quality and high heterogeneity imply that a cautious interpretation of this conclusion is warranted. Although those who exercise at high volumes may accumulate more CAC, the resulting plaques may be more calcified and stable. Altered plaque composition and increased cardiorespiratory fitness likely contribute to lower mortality among high-volume exercisers compared to less-active cohorts, despite their having similar CAC scores; however, the influence of exercise volume on CV event rates has become less clear in light of emerging evidence. More research is required to understand the clinical implications of elevated CAC scores in high-volume exercisers. Future studies should use randomized designs to isolate the effects of exercise volume. In addition, future research should aim to investigate the influence of exercise intensity on CAC. Standardized volume categories and reporting of CAC in absolute terms, supplemented by relative values, would improve consistency and generalizability.

## References

[1] Ekelund U., Tarp J., Steene-Johannessen J. (2019). Dose-response associations between accelerometry measured physical activity and sedentary time and all cause mortality: systematic review and harmonised meta-analysis. BMJ.

[2] Nocon M., Hiemann T., Müller-Riemenschneider F. (2008). Association of physical activity with all-cause and cardiovascular mortality: a systematic review and meta-analysis. Eur J Cardiovasc Prev Rehabil.

[3] Pedisic Z., Shrestha N., Kovalchik S. (2020). Is running associated with a lower risk of all-cause, cardiovascular and cancer mortality, and is the more the better? A systematic review and meta-analysis. Br J Sports Med.

[4] Aengevaeren V.L., Mosterd A., Bakker E.A. (2023). Exercise volume versus intensity and the progression of coronary atherosclerosis in middle-aged and older athletes: findings from the MARC-2 study. Circulation.

[5] Aengevaeren V.L., Mosterd A., Braber T.L. (2017). Relationship between lifelong exercise volume and coronary atherosclerosis in athletes. Circulation.

[6] De Bosscher R., Dausin C., Claus P. (2023). Lifelong endurance exercise and its relation with coronary atherosclerosis. Eur Heart J.

[7] Laddu D.R., Rana J.S., Murillo R. (2017). 25-year physical activity trajectories and development of subclinical coronary artery disease as measured by coronary artery calcium: the Coronary Artery Risk Development in Young Adults (CARDIA) Study. Mayo Clin Proc.

[8] Merghani A., Maestrini V., Rosmini S. (2017). Prevalence of subclinical coronary artery disease in Masters endurance athletes with a low atherosclerotic risk profile. Circulation.

[9] Pavlovic A., DeFina L.F., Leonard D. (2024). Coronary artery calcification and high-volume physical activity: role of lower intensity vs. longer duration of exercise. Eur J Prev Cardiol.

[10] Shuval K., Leonard D., DeFina L.F. (2024). Physical activity and progression of coronary artery calcification in men and women. JAMA Cardiol.

[11] Hsu J.J., Tintut Y., Demer L.L. (2025). Paradox of exercise and coronary artery calcification: potential underlying mechanisms. Circ Res.

[12] Mori H., Torii S., Kutyna M. (2018). Coronary artery calcification and its progression: What does it really mean?. JACC Cardiovasc Imaging.

[13] Criqui M.H., Denenberg J.O., Ix J.H. (2014). Calcium density of coronary artery plaque and risk of incident cardiovascular events. JAMA.

[14] Budoff M.J., Shaw L.J., Liu S.T. (2007). Long-term prognosis associated with coronary calcification. J Am Coll Cardiol.

[15] Aengevaeren V.L., Mosterd A., Sharma S. (2020). Exercise and coronary atherosclerosis: observations, explanations, relevance, and clinical management. Circulation.

[16] Zambrano A., Tintut Y., Demer L.L., Hsu J.J. (2023). Potential mechanisms linking high-volume exercise with coronary artery calcification. Heart.

[17] Parry-Williams G., Sharma S. (2020). The effects of endurance exercise on the heart: panacea or poison?. Nat Rev Cardiol.

[18] Baggish A.L., Levine B.D. (2017). Coronary artery calcification among endurance athletes: "hearts of stone.". Circulation.

[19] Agatston A.S., Janowitz W.R., Hildner F.J. (1990). Quantification of coronary artery calcium using ultrafast computed tomography. J Am Coll Cardiol.

[20] Herrmann S.D., Willis E.A., Ainsworth B.E. (2024). 2024 adult compendium of physical activities: a third update of the energy costs of human activities. J Sport Health Sci.

[21] Ross R., Chaput J.P., Giangregorio L.M. (2020). Canadian 24-hour movement guidelines for adults aged 18-64 years and adults aged 65 years or older: an integration of physical activity, sedentary behaviour, and sleep. Appl Physiol Nutr Metab.

[22] Budoff M.J., Young R., Burke G. (2018). Ten-year association of coronary artery calcium with atherosclerotic cardiovascular disease (ASCVD) events: the Multi-Ethnic Study of Atherosclerosis (MESA). Eur Heart J.

[23] Bertoni A.G., Whitt-Glover M.C., Chung H. (2009). The association between physical activity and subclinical atherosclerosis: the Multi-Ethnic Study of Atherosclerosis. Am J Epidemiol.

[24] Sung K.C., Hong Y.S., Lee J.Y. (2021). Physical activity and the progression of coronary artery calcification. Heart.

[25] Yoo T.K., Lee S.H., Rhim H.C. (2023). Association of cardiovascular mortality with concurrent coronary artery calcification and physical activity: a cohort study. Medicina (Kaunas).

[26] DeFina L.F., Radford N.B., Barlow C.E. (2019). Association of all-cause and cardiovascular mortality with high levels of physical activity and concurrent coronary artery calcification. JAMA Cardiol.

[27] Gao J.W., Hao Q.Y., Lu L.Y. (2022). Associations of long-term physical activity trajectories with coronary artery calcium progression and cardiovascular disease events: results from the CARDIA study. Br J Sports Med.

[28] Jafar O., Friedman J., Bogdanowicz I. (2019). Assessment of coronary atherosclerosis using calcium scores in short- and long-distance runners. Mayo Clin Proc Innov Qual Outcomes.

[29] Möhlenkamp S., Lehmann N., Breuckmann F. (2008). Running: the risk of coronary events: prevalence and prognostic relevance of coronary atherosclerosis in marathon runners. Eur Heart J.

[30] Berry J.D., Zabad N., Kyrouac D. (2025). High-volume physical activity and clinical coronary artery disease outcomes: findings from the Cooper Center Longitudinal Study. Circulation.

[31] Desai M.Y., Nasir K., Rumberger J.A. (2004). Relation of degree of physical activity to coronary artery calcium score in asymptomatic individuals with multiple metabolic risk factors. Am J Cardiol.

[32] Feuchtner G., Langer C., Barbieri F. (2019). Relationship of exercise to coronary artery disease extent, severity and plaque type: a coronary computed tomography angiography study. J Cardiovasc Comput Tomogr.

[33] Feuchtner G.M., Langer C., Senoner T. (2020). Differences in coronary vasodilatory capacity and atherosclerosis in endurance athletes using coronary CTA and computational fluid dynamics (CFD): comparison with a sedentary lifestyle. Eur J Radiol.

[34] Gerber Y., Gabriel K.P., Jacobs D.R. (2025). The relationship of cardiorespiratory fitness, physical activity, and coronary artery calcification to cardiovascular disease events in CARDIA participants. Eur J Prev Cardiol.

[35] Hamer M., Venuraju S.M., Lahiri A., Rossi A., Steptoe A. (2012). Objectively assessed physical activity, sedentary time, and coronary artery calcification in healthy older adults. Arterioscler Thromb Vasc Biol.

[36] German C.A., Fanning J., Singleton M.J. (2022). Physical activity, coronary artery calcium, and cardiovascular outcomes in the Multi-Ethnic Study of Atherosclerosis (MESA). Med Sci Sports Exerc.

[37] Thomas I.C., Takemoto M.L., Forbang N.I. (2020). Associations of recreational and non-recreational physical activity with coronary artery calcium density vs. volume and cardiovascular disease events: the Multi-Ethnic Study of Atherosclerosis. Eur Heart J Cardiovasc Imaging.

[38] Papatheodorou E., Aengevaeren V.L., Eijsvogels T.M.H. (2024). Prevalence of coronary atherosclerosis in female Masters endurance athletes. Circulation.

[39] Bhatia H.S., Lin F., Thomas I.C. (2022). Coronary artery calcium incidence and changes using direct plaque measurements: the MASALA study. Atherosclerosis.

[40] Braber T.L., Mosterd A., Prakken N.H. (2016). Occult coronary artery disease in middle-aged sportsmen with a low cardiovascular risk score: the Measuring Athlete's Risk of Cardiovascular Events (MARC) Study. Eur J Prev Cardiol.

[41] Berge K., Janssen S., Velthuis B.K. (2025). Predictors of coronary atherosclerosis in middle-aged and older athletes: the MARC-2 Study. Eur Heart J Cardiovasc Imaging.

[42] Janssen S.L.J.E., Aengevaeren V.L., De Vries F. (2025). Exercise-induced changes in hemodynamics, hormones, electrolytes, and inflammatory markers in veteran athletes with and without coronary atherosclerosis. Med Sci Sports Exerc.

[43] Tsiflikas I., Thomas C., Fallmann C. (2015). Prevalence of subclinical coronary artery disease in middle-aged, male marathon runners detected by cardiac CT. Rofo.

[44] Nassenstein K., Breuckmann F., Lehmann N. (2009). Left ventricular volumes and mass in marathon runners and their association with cardiovascular risk factors. Int J Cardiovasc Imaging.

[45] Kleiven Ø., Bjørkavoll-Bergseth M.F., Omland T. (2020). Endurance exercise training volume is not associated with progression of coronary artery calcification. Scand J Med Sci Sports.

[46] Dores H., de Araújo Gonçalves P., Monge J. (2020). Subclinical coronary artery disease in veteran athletes: Is a new preparticipation methodology required?. Br J Sports Med.

[47] Roberts W.O., Schwartz R.S., Kraus S.M. (2017). Long-term marathon running is associated with low coronary plaque formation in women. Med Sci Sports Exerc.

[48] Abdelaziz A., Elshahat A., Gadelmawla A.F. (2025). Sex differences in the impact of exercise volume on subclinical coronary atherosclerosis: a meta-analysis. JACC Adv.

[49] McClelland R.L., Chung H., Detrano R., Post W., Kronmal R.A. (2006). Distribution of coronary artery calcium by race, gender, and age: results from the Multi-Ethnic Study of Atherosclerosis (MESA). Circulation.

[50] Radford N.B., DeFina L.F., Leonard D. (2018). Cardiorespiratory fitness, coronary artery calcium, and cardiovascular disease events in a cohort of generally healthy middle-age men: results from the Cooper Center Longitudinal Study. Circulation.

[51] Nguyen P.K., Terashima M., Fair J.M. (2011). Physical activity in older subjects is associated with increased coronary vasodilation. JACC Cardiovasc Imaging.

[52] Haskell W.L., Sims C., Myll J. (1993). Coronary artery size and dilating capacity in ultradistance runners. Circulation.

[53] Laughlin M.H., Bowles D.K., Duncker D.J. (2012). The coronary circulation in exercise training. Am J Physiol Heart Circ Physiol.

[54] Budoff M.J., Hokanson J.E., Nasir K. (2010). Progression of coronary artery calcium predicts all-cause mortality. JACC Cardiovasc Imaging.

[55] Yong Y., Giovannucci J., Pang S.N. (2025). Coronary artery calcium density and risk of cardiovascular events: a systematic review and meta-analysis. JACC Cardiovasc Imaging.

[56] van Rosendael A.R., van den Hoogen I.J., Gianni U. (2021). Association of statin treatment with progression of coronary atherosclerotic plaque composition. JAMA Cardiol.

[57] Puri R., Nicholls S.J., Shao M. (2015). Impact of statins on serial coronary calcification during atheroma progression and regression. J Am Coll Cardiol.

[58] Claessen G., Eijsvogels T.M.H., Albert C.M. (2025). Coronary atherosclerosis in athletes: emerging concepts and preventive strategies. Eur Heart J.

[59] Sung D.E., Sung K.C. (2024). The paradox of physical activity and coronary artery calcification: implications for cardiovascular risk. J Clin Med.

