# Supplementary material for: Exercise Volume and Coronary Artery Calcification: A Systematic Review
Source: CJC Open. 2025 Dec 30;8(4):457–65. doi: 10.1016/j.cjco.2025.12.009 (PMC13084289; doi:10.1016/j.cjco.2025.12.009)

**Supplemental Figure S1**. Meta-regression bias analysis funnel plot

**
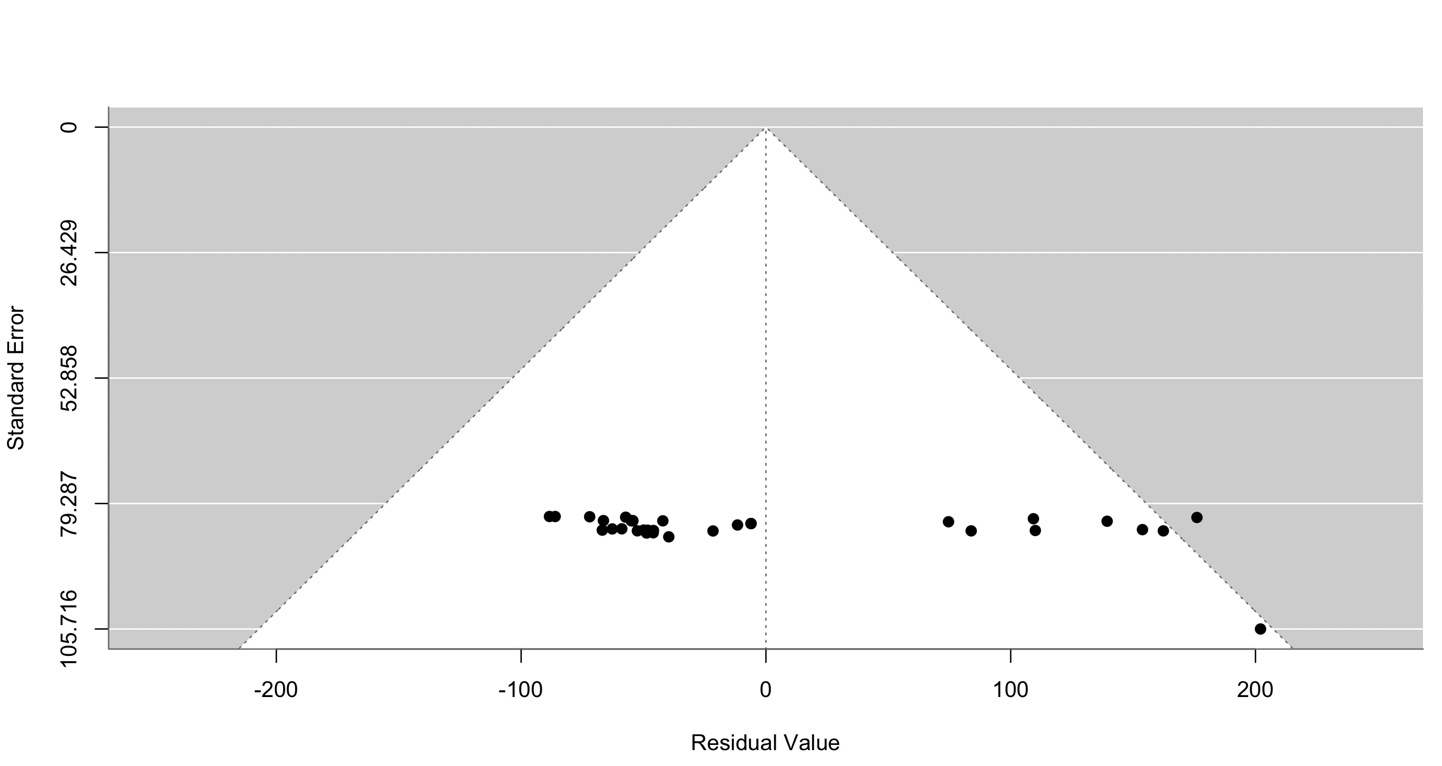
**

**Methodology for meta-regression:** To associate the exercise volume as the primary predictor of the CAC score as the primary outcome, we planned to conduct a meta-regression using the “metafor” package in RStudio environment version 2021.09.1 (RStudio, PBC). Studies reporting CAC scores as absolute values were to be included, with each subgroup of exercise volume within a study representing an individual dataset within the meta-regression. To standardize the CAC values, all studies providing median and interquartile ranges were converted to means and standard deviations. A random effects model was used with the assumption of large heterogeneity on the study participants and methodologies, implying that the true effect of exercise volume and CAC score can potentially differ from study to study. To verify this, the I² statistics and visual funnel plot analysis were also done to assess heterogeneity, alongside calculation of Egger’s test. The meta-regression coefficient was extracted from the model to estimate the estimated average change in CAC score for a one-unit increase in exercise volume. Statistical significance was set at p<0.05. A multivariable meta-regression model, adjusting for age for each group, was a part of the planned analysis.

**Supplemental Table S1.** Summary of articles meeting inclusion criteria.

| Reference | Population | Study type | Sex | Sample Size | Age^a^ | Confounder adjustments | Exercise Volume^a^ (min/wk) | CAC Score^a^ - Absolute | CAC Score^a^ - Relative | Mortality or CV events |
| --- | --- | --- | --- | --- | --- | --- | --- | --- | --- | --- |
| Desai et al. (2004) | Asymptomatic patients referred for cardiac risk assessment w/ CAC | Cross-sectional | M & F | 492 | a) M=54, F=57  b) M=52, F=56 | Age, sex, obesity, HTN, DLD, smoking, and FHx of premature CAD. | a) <60 (n=205)  b) >90 (n=287) | a) 18  b) 11 | % of population…  a) CAC 0 = 24%, CAC 1-100 = 27%, CAC 101-400 = 20%, CAC >400 = 38%  b) CAC 0 = 45%, CAC 1-100 = 36%, CAC 101-400 = 39%, CAC >400 = 17%) | Not reported |
| Mohlenkamp et al. (2008) | M athletes >50 yrs who have run at least 5 full-distance marathons during the previous 3 yrs and matched controls from the Heinz Nixdorf Recall Study | Cross-sectional | M | 1188 | a) 57.2  b) 57.1  c) 57.2 | n/a | a) 163  b) 206  c) 551 | a) 38  b) 12  c) 36 | % of population…  a) CAC of 0 = 18.4%, >75th percentile = 24.2%, 0 to <10 =34.61%, 10 to <100 = 29.05%, 100 to <400 = 22.80%, >400 = 13.54  b) CAC of 0 = 31.5%, >75th percentile = 14.8%, 0 to <10 =48.61%, 10 to <100 = 29.63%, 100 to <400 = 13.43%, >400 = 8.33  c) CAC of 0 = 28.7%, >75th percentile = 25.0%, 0 to <10 =40.74%, 10 to <100 = 23.15%, 100 to <400 = 23.15%, >400 = 12.96 | No mortality in the marathon runners over 21 months of follow-up. However, 4 runners had cardiac events: 2 occurrences of VT during exercise, 3 underwent PCI, and 2 had CABG. |
| Bertoni et al. (2009) | 45-84 yrs old M & F in the MESA Study | Cross-sectional | M & F | 6814 | M=62.3  F=62.4 | Age, race/ethnicity, smoking, BMI, HTN, SBP, DM, total cholesterol, HDL-C, clinic site, education, income. | a) 7-99  b) 106-205  c) >212 | Not reported | % of population CAC >0:  a) 39.4%  b) 37.9%  c) 41.1% | Not reported |
| Nassenstein et al. (2009) | M >50 yrs who have run at least 5 full distance marathons in the previous 3 yrs | Cross-sectional | M | 108 | 57.2 | n/a | 393 | 36 | % of population with CAC <100 = 62.9%, 100 to <400 = 23.8%, >400 = 13.3%) | Not reported |
| Hamer et al. (2012) | Whitehall II epidemiologic cohort without known CVD | Cross-sectional | M & F | 443 | a) 68.7  b) 66.8  c) 64.9 | Age, sex, statins use, smoking, SBP, HDL-C, TG, HbA1c, and BMI. | a) 32  b) 134  c) 382 | a) 13.9  b) 17.9  c) 11.1 | Not reported | Not reported |
| Tsiflikas et al. (2015) | M marathon runners > 45 yrs old | Cross-sectional | M | 50 | 52.6 | n/a | 300 | 43.5 | % of population CAC 0 = 52%, 1-10 = 18%, 10-100 = 18%, 100-400 = 8%, >400 = 2% | Not reported |
| Braber et al. (2016) | Asymptomatic/healthy M >45 yrs engaged in sport (MARC Study) | Cross-sectional | M | 318 | 54.7 | n/a | 180 | 0 (45-54yrs, n=176)  16 (55-64yrs, n=113)  39 (65-79yrs, n=29) | % of population CAC 0 = 47.5%, 1-99 = 36.2%, 100-399 = 9.1%, >400 = 7.2% | Not reported |
| Aengevaeren et al. (2017) | Asymptomatic/healthy M >45 yrs engaged in sport (MARC Study) | Cross-sectional | M | 284 | a) 54.4  b) 54.8  c) 55.9 | BMI, SBP, smoking, antiHTN medication, DLD, FHx of CAD, statin use, and DM. | a) 90  b) 180  c) 342 | a) 0  b) 0.8  c) 9.4 | % of population CAC = 0  a) 57%  b) 50%  c) 32% | Not reported |
| Laddu et al. (2017) | M & F 18-30 yrs old at baseline followed for 25 yrs (CARDIA Study) | Cross-sectional | M & F | 3175 | 50.4 | Age, race, sex, smoking,  DM, DLD, education, BMI, and HTN. | a) <150  b) 150-450  c) >450 | Not reported | % of population CAC >0, >20, >100  a) 29.0%, 16.6%, 8.6%  b) 33.2%, 21.4%, 10.5%  c) 41.8%, 25.0%, 11.9% | Not reported |
| Merghani et al. (2017) | Masters athletes in the United Kingdom who were recruited from elite running and cycling clubs. Healthy controls engaged in exercise were also recruited | Cross-sectional | M & F | 244 | M:  a) 52.5  b) 55.1  F:  a) 54.2  b) 53.1 | All variables  associated with CAD in univariable analyses and a  compound of age and years of training. | M:  a) 114  b) 450  F:  a) 114  b) 462 | Median CAC = 0  Those w/ CAC >0 (athletes, n=25):  M:  a) 3  b) 86  F:  a) 7  b) 7 | % of population CAC >50th percentile, >70th percentile, >0, >10, >100, >300, >400  M:  a) 22.2%, 14.8%, 40.7%, 18.5%, 7.4%, 0%, 0%  b) 27.4%, 15.1%, 48.1%, 41.5%, 18.9%, 11.3%, 7.5%  F:  a) 31.6%, 26.4%, 31.6%, 21%, 10.5%, 5.2%, 2.6%  b) 21.7%, 19.6%, 21.7%, 10.9%, 6.5%, 4.3%, 2.2% | Not reported |
| Roberts et al. (2017) | F who ran at least 1 marathon per yr in the previous 10-25 yrs | Cross-sectional | F | 26 | 56 | n/a | a) 678  b) 644 | a) CAC = 0 (n=21)  b) CAC =1.4 (n=5) | Not reported | Not reported |
| Budoff et al. (2018) | M & F 45-84 yrs old free of CVD (MESA Study) | Prospective cohort | M & F | 6814 | a) 62.1  b) 62.4  c) 61.3  d) 62.6 | Age, race, sex, education, income, smoking, LDL-C, HDL-C, DM, lipid-lowering medications, SBP, antiHTN medication, physical activity, and BMI. | a) 95  b) 86  c) 74  d) 124 | CAC score, if non-zero:  a) 282.6  b) 188.8  c) 274.6  d) 331.4 | % with CAC, CAC 0, CAC 1-100, CAC 101-300, CAC 300+  a) 43.5, 3.9, 7.7, 14.7, 24.5  b) 50.2, 1.3, 4.7, 8.3, 13.1  c) 45.2, 3.1, 10.3, 19.1, 21.7  d) 57.0, 2.4, 7.3, 10.8, 16.2 | Not reported |
| DeFina et al. (2019) | 40-80yrs old, who were evaluated at the study clinic and had a history of exercise and a CAC scan | Prospective cohort | M & F | 31259  (M = 21758; F = 9501) | M: CAC <100  a) 49.5  b) 49.2  c) 50.0  M: CAC >100  a) 58.7  b) 57.9  c) 58.9  F: CAC <100  a) 52.3  b) 50.6  c) 50.6  F: CAC >100  a) 62.5  b) 62.3  c) 60.5 | Age, smoking, BMI, blood glucose, DLD, and SBP. | M: CAC <100  a) 104  b) 334  c) 713  M: CAC >100  a) 105  b) 354  c) 767  F: CAC <100  a) 120  b) 387  c) 725  F: CAC >100  a) 94  b) 395  c) 766 | M: CAC <100  a) 11.3  b) 10.3  c) 11.5  M: CAC >100  a) 736.1  b) 680.9  c) 806.7  F: CAC <100  a) 4.6  b) 3.2  c) 4.5  F: CAC >100  a) 442.4  b) 375.3  c) 315.4 | Not reported | After 10 years of follow-up, high-volume exercise is not associated with all-cause or CV mortality. |
| Feuchtner et al. (2019) | Patients >21 yrs at low-intermediate risk of CAD referred for coronary CTA | Cross-sectional | M & F | 155 | a) 55  b) 52 | n/a | a) <60  b) >180 | a) 57.1  b) 27.2 | Not reported | No MACE observed after 1 year of follow-up. |
| Jafar et al. (2019) | >45 yr old runners who have run competitively for >10 yrs | Cross-sectional | M & F | 56 | a) 59.3  b) 53.0  c) 60.9 | Age, sex, and number of years running. | a) 385  b) 460  c) 493 | Not reported | % of population CAC >0, >50^th^ percentile, >100, F >50^th^ percentile  a) 23.1%, 19.2%, 11%, 7.7%  b+c) 73.3%, 70%, 33%, 71.4% | Not reported |
| Dores et al. (2020) | M >40 yr old who have engaged in regular exercise for >4 hr/wk for >5 yrs | Cross-sectional | M | 105 | 48 | n/a | 466 | Median CAC = 0  In those w/ CAC >0 (n=44): 34 | % of population CAC 0 = 59%, 1-10 = 16.2%, 10-100 = 15.2%, >100 = 9.5% | Not reported |
| Feuchtner et al. (2020) | Endurance athletes who underwent coronary CTA for clinical indications | Cross-sectional | M & F | 52 | 54.4 | n/a | >180 | 29.9 | Not reported | Not reported |
| Kleiven et al. (2020) | >16 yr old Norwegian athletes participating in the 91-km mountain bike race | Prospective cohort | M & F | 61 | CAC 0: 44.3  CAC >0: 52.9 | Age, sex, LDL, BMI, SBP, exercise volume, baseline CAC score. | 395 | CAC 0 (n=46): 0  CAC >0 (n=15): 40 | Not reported | Not reported |
| Thomas et al. (2020) | 45-84 yr old M & F in the MESA Study | Cross-sectional | M & F | 3393 | a) 66.3  b) 66.9  c) 66.2  d) 65.8 | Age, sex, race, income, education, SBP, antiHTN medication, total cholesterol, HDL-C, statin use, smoking, DM, and BMI. | a) 43  b) 116  c) 218  d) 466 | a) 71  b) 99  c) 100  d) 95 | Not reported | Recreational exercise was inversely associated with MACE. |
| German et al. (2021) | 45-84 yr old M & F in the MESA Study | Prospective cohort | M & F | 6777 | CAC <100  a) 60.0  b) 60.1  c) 60.1  d) 60.0  CAC >100  a) 69.3  b) 69.8  c) 68.9  d) 68.1 | Age, sex, race, BMI, smoking, SBP, DBP, DM, antiHTN medication, total cholesterol, HDL-C, LDL-C, TG, and statin use. | CAC <100  a) 1  b) 56  c) 158  d) 516  CAC >100  a) 1  b) 58  c) 160  d) 517 | CAC <100  a) 11.4  b) 10.3  c) 10.7  d) 11.9  CAC >100  a) 563.5  b) 633.3  c) 588.0  d) 558.7 | Not reported | High levels of exercise were associated with decreased all-cause mortality. |
| Sung et al. (2021) | M & F >30 yrs old who completed a PA questionnaire and had >2 CAC scans | Prospective cohort | M & F | 13565 | a) 41.7  b) 43.2 | Age, sex, center smoking, alcohol,  education, FHx of CVD, BMI, SBP, LDL-C, HDL-C, TG, fasting glucose, HTN, DM, and lipid-lowering therapy. | a) >71  b) >353 | a) 14.3  b) 24.3 | % of population CAC >0, 1-99, >100  a) 21.7%, 18.1%, 3.5%  b) 25.4%, 20.2%, 5.0% | Not reported |
| Bhatia et al. (2022) | M & F 40-84 yrs old who completed two exams, and had CAC score >5 on at least one assessment (MASALA Study) | Prospective cohort | M & F | 387 | 62.8 | Age, sex, baseline CAC volume and density, time between exams, HTN, DM, smoking, total cholesterol, HDL-C, exercise, statin use, Lp(a), and fasting insulin. | 148 | Not reported | % CAC 1-100, 101-400, >400: 48.1%, 25.8%, 26.1% | Not reported |
| Gao et al. (2022) | M & F 18-30 yrs old at baseline followed for 15 yrs (CARDIA Study) | Prospective cohort | M & F | 2497 | 40.4 | Age, alcohol, antiHTN medication, BMI, DM, education, LDL-C, race, serum creatinine, SBP, and smoking. | a) <150  b) 150-450  c) >450 | Not reported | % of population CAC = 0, 0-100, >100  a) 91.4%, 7.1%, 1.4%.  b) 91.2%, 7.6%, 1.2%  c) 85.8%, 13.0%, 1.2% | MACE was not different between the 3 categories of exercise volume. |
| Aengevaeren et al. (2023) | Asymptomatic M >45 yrs old who had abnormalities on sports medical exam (MARC-2 Study) | Prospective cohort | M | 289 | 60 | Age, BMI, SBP, smoking, total cholesterol, FHx of CAD antiHTN/DM/statin medications use, CAC score or number of plaques at baseline, and time between CT scans. | 186 | 31 | % of population CAC = 0: baseline = 52%, follow-up = 71%.  In those w/ CAC >0:  CAC >100 - baseline = 15%, follow-up = 31%; CAC >400 - baseline = 6%, follow-up = 13%; CAC >1000 - baseline = 1%, follow-up = 6% | Not reported |
| De Bosscher et al. (2023) | M 45-70 yrs old consisting of endurance athletes and non-athletes (<3 hr/wk) (Master@Heart Study) | Cross-sectional | M | 558 | a) 55  b) 55  c) 56 | Age, BMI, SBP, DBP, total cholesterol, LDL-C, HbA1c, and FHx of CAD. | a) 60  b) 600  c) 660 | a) 0  b) 1.3  c) 8.5 | CAC Score Percentile:  a) 0  b) 23rd  c) 44th  % of population CAC >0, >10, >100, >400  a) 45.5%, 33.5%, 14.8%, 4%  b) 52.4%, 37.7%, 16.2%, 4.7%  c) 57.1%, 49.2%, 23%, 5.2% | Not reported |
| Yoo et al. (2023) | >18 yr old who completed health exams, PA questionnaires, and CAC scans (Kangbuk Samsung Health Study) | Prospective cohort | M & F | 30857 | a) 41.2  b) 43.9 | Sex, age, BMI, alcohol, smoking, education,  SBP, fasting glucose, LDL, and Hx of CVD/DM/ DLD/HTN. | a) >71  b) >353 | Not reported | % of population CAC = 0, >0  a) 83.84%, 16.16%  b) 78.22%, 21.78% | Individuals with CAC had higher CV mortality, with no association to exercise volume. Higher volume appeared protective in those with no CAC. |
| Pavlovic et al. (2024) | M 40-80yrs old, who were evaluated at the study clinic and had a history of exercise and a CAC scan (Cooper Center Longitudinal Study) | Cross-sectional | M & F | 33637 | a) 50.6  b) 51.4  c) 52.4 | Age, smoking, BMI, glucose, cholesterol, and SBP. | M: n=23383  a) 70  b) 186  c) 485  F: n=10254  a) 69  b) 191  c) 470 | M:  a) 143.3  b) 159.8  c) 199.7  F:  a) 26.6  b) 27.7  c) 20.4 | % of population CAC >100  M:  a) 19.6%  b) 22.2%  c) 26.4%  F:  a) 5.8%  b) 5.7%  c) 5% | Not reported |
| Shuval et al. (2024) | >40 yrs old who had >2 health exams and completed PA questionnaires and CAC scans (Cooper Center Longitudinal Study) | Prospective cohort | M & F | 8771 | M=58  F=58.8 | Age, baseline CAC, type of CAC scanner, smoking, BMI, fasting glucose, total cholesterol, SBP, and statin use. | a) <176  b) 176-353  c) >353 | Not reported | % of population CAC = 0, 1-99, >100  M:  a) 51.3%, 30.6%, 18.1%  b) 50.2%, 29.3%, 20.5%  c) 46.9%, 30.2%, 22.9%  F:  a) 79.4%, 14.7%, 5.9%  b) 79.7%, 16.2%, 4.1%  c) 79.3%, 17.3%, 3.3% | Not reported |
| Papatheodorou et al. (2024) | F Masters athletes >40 yrs who have exercised for >10yrs and competed in >10 endurance events. Controls were of similar age. Those with pre-existing CVD were excluded. | Cross-sectional | F | 255 | a) 54  b) 54.8 | n/a | a) 27  b) 480 | a) 0  b) 0 | % of population CAC >0, >100, >400, >50^th^ percentile, >75^th^ percentile  a) 32%, 8.5%, 3%, 32%, 25%  b) 21%, 3.6%, 1%, 19%, 14% | Not reported |
| Berge et al. (2025) | >45 yrs engaged in competitive or recreational sports without established CVD (MARC-2 Study) | Cross-sectional | M | 289 | 60 | Age, BMI, SBP, smoking, LDL-C, FHx, DM | 186 | 31 | Not reported | Not reported |
| Berry et al. (2025) | Adults without known CAD, self-referred or refereed by employer for preventative care (Cooper Center Longitudinal Study) | Cross-sectional | M & F | 14071 | 54 | n/a | a) 24  b) 162  c) 306  d) 630 | a) 208.1  b) 238.3  c) 237.0  d) 266.5 | % of population CAC >100  a) 28.5%  b) 30.4%  c) 30.5%  d) 34.5% | Mortality was lowest in the highest exercise volume group.  Lowest risk of CV events was in the intermediate exercise volume group. |
| Gerber et al. (2025) | M & F 18-30 yrs old at baseline followed for 20 yrs (CARDIA Study) | Prospective cohort | M & F | 3128 | 45.3 | Age, sex, race, study center, education, Framingham risk score, total cholesterol, HDL-C, SBP, BMI, DM, smoking, lipid lowering therapy, antiHTN therapy | a) <150  b) >150 | Not reported | % of population CAC >0  a) 19.7%  b) 20.6% | Higher exercise volume was associated with lower CV event risk and mortality in those without CAC.  However, the presence of any CAC conferred a CV event risk that was not mitigated by exercise volume. |
| Janssen et al. (2025) | Participants from the MARC-2 Study invited for follow-up study | Cross-sectional | M | 58 | 61 | n/a | 168 | 185 | % of population CAC >300 = 36.2% | Not reported |

^a^ Lowercase letters (a, b, etc.) represent distinct exercise volume subgroups within each study. Abbreviations: BMI = body mass index; CABG = coronary artery bypass grafting; CAC = coronary artery calcium; CAD = coronary artery disease; CTA = computed tomography angiogram; CV = cardiovascular; CVD = cardiovascular disease; DBP = diastolic blood pressure; DLD = dyslipidemia; DM = diabetes mellitus; F = female; FHx = family history; HbA1c = glycated hemoglobin; HDL-C = high-density lipoprotein cholesterol; HTN = hypertension; LDL-C = low-density lipoprotein cholesterol; Lp(a) = lipoprotein(a); M = male; MACE = major adverse cardiovascular events; PA = physical activity; PCI = percutaneous coronary intervention; SBP = systolic blood pressure; TG = triglycerides; VT = ventricular tachycardia; Yrs = years, Yr = year

**Supplemental Table S2:** MINORS bias checklist

|  | **A clearly stated aim** | **Inclusion of consecutive patients** | **Prospective collection of data** | **Endpoints appropriate to the aim of the study** | **Unbiased assessment of the study endpoint** | **Follow-up period appropriate to the aim of the study** | **Loss to follow up less than 5%** | **Prospective calculation of the study size** | **An adequate control group** | **Contemporary groups** | **Baseline equivalence of groups** | **Adequate statistical analyses** |
| --- | --- | --- | --- | --- | --- | --- | --- | --- | --- | --- | --- | --- |
| **Non-comparative (n=8)** | | | | | | | | | | | | |
| Nassenstein et al (2009) | 2 | 2 | 1 | 2 | 0 | 0 | 0 | 0 | -- | -- | -- | -- |
| Tsiflikas et al. (2015) | 2 | 2 | 1 | 2 | 0 | 0 | 0 | 0 | -- | -- | -- | -- |
| Braber et al. (2016) | 2 | 2 | 1 | 2 | 2 | 2 | 0 | 1 | -- | -- | -- | -- |
| Dores et al. (2020) | 2 | 2 | 2 | 2 | 0 | 0 | 0 | 0 | -- | -- | -- | -- |
| Kleiven et al. (2020) | 2 | 2 | 2 | 2 | 0 | 2 | 1 | 0 | -- | -- | -- | -- |
| Aengevaeren et al. (2023) | 2 | 2 | 2 | 2 | 0 | 2 | 2 | 2 | -- | -- | -- | -- |
| Berge et al. (2025) | 2 | 1 | 2 | 2 | 0 | 0 | 0 | 0 | -- | -- | -- | -- |
| Janssen et al. (2025) | 2 | 1 | 2 | 2 | 1 | 0 | 0 | 0 | -- | -- | -- | -- |
| **Comparative (n=25)** | | | | | | | | | | | | |
| Desai et al. (2004) | 2 | 2 | 2 | 2 | 2 | 0 | 0 | 0 | 2 | 2 | 2 | 1 |
| Mohlenkamp et al. (2008) | 2 | 2 | 2 | 2 | 0 | 1 | 1 | 0 | 2 | 0 | 1 | 2 |
| Bertoni et al. (2009) | 2 | 1 | 2 | 1 | 0 | 0 | 0 | 0 | 2 | 2 | 0 | 2 |
| Hamer et al. (2011) | 2 | 1 | 1 | 2 | 2 | 0 | 0 | 0 | 2 | 2 | 0 | 2 |
| Aengevaeren et al. (2017) | 2 | 2 | 2 | 2 | 2 | 0 | 0 | 0 | 2 | 2 | 0 | 2 |
| Laddu et al. (2017) | 2 | 2 | 2 | 2 | 0 | 1 | 0 | 0 | 2 | 2 | 1 | 2 |
| Merghani et al. (2017) | 2 | 2 | 2 | 2 | 2 | 0 | 0 | 0 | 2 | 2 | 2 | 2 |
| Roberts et al. (2017) | 2 | 2 | 2 | 2 | 0 | 0 | 0 | 0 | 2 | 0 | 2 | 2 |
| Budoff et al. (2018) | 2 | 2 | 2 | 2 | 2 | 2 | 1 | 0 | 2 | 2 | 0 | 2 |
| DeFina et al. (2019) | 2 | 2 | 2 | 2 | 0 | 2 | 1 | 0 | 2 | 2 | 0 | 2 |
| Feuchtner et al. (2019) | 2 | 2 | 2 | 2 | 0 | 2 | 0 | 0 | 2 | 2 | 2 | 2 |
| Jafar et al. (2019) | 2 | 2 | 2 | 1 | 0 | 0 | 0 | 0 | 2 | 2 | 0 | 1 |
| Feuchtner et al. (2020) | 2 | 1 | 2 | 2 | 0 | 0 | 0 | 0 | 2 | 2 | 2 | 2 |
| Thomas et al. (2020) | 2 | 2 | 2 | 2 | 0 | 2 | 2 | 0 | 2 | 2 | 0 | 2 |
| German et al. (2021) | 2 | 2 | 2 | 2 | 2 | 2 | 1 | 0 | 2 | 2 | 0 | 2 |
| Sung et al. (2021) | 2 | 2 | 2 | 2 | 0 | 1 | 1 | 1 | 2 | 2 | 0 | 2 |
| Bhatia et al. (2022) | 2 | 2 | 2 | 2 | 2 | 2 | 2 | 0 | 2 | 0 | 0 | 2 |
| Gao et al. (2022) | 2 | 2 | 2 | 2 | 2 | 2 | 1 | 0 | 2 | 2 | 0 | 2 |
| De Bosscher et al. (2023) | 2 | 2 | 2 | 2 | 2 | 2 | 2 | 2 | 2 | 2 | 2 | 2 |
| Yoo et al. (2023) | 2 | 2 | 2 | 2 | 0 | 2 | 0 | 0 | 2 | 2 | 0 | 1 |
| Pavlovic et al. (2024) | 2 | 2 | 1 | 1 | 0 | 0 | 0 | 0 | 2 | 2 | 1 | 2 |
| Shuval et al. (2024) | 2 | 2 | 2 | 2 | 0 | 2 | 1 | 1 | 2 | 2 | 1 | 2 |
| Papatheodorou et al. (2024) | 2 | 1 | 2 | 2 | 0 | 0 | 0 | 0 | 2 | 2 | 1 | 1 |
| Berry et al. (2025) | 2 | 1 | 2 | 2 | 1 | 2 | 1 | 0 | 2 | 2 | 1 | 2 |
| Gerber et al. (2025) | 2 | 1 | 2 | 2 | 1 | 2 | 0 | 0 | 2 | 2 | 1 | 2 |

**Supplemental Appendix S1**: PRISMA Checklist
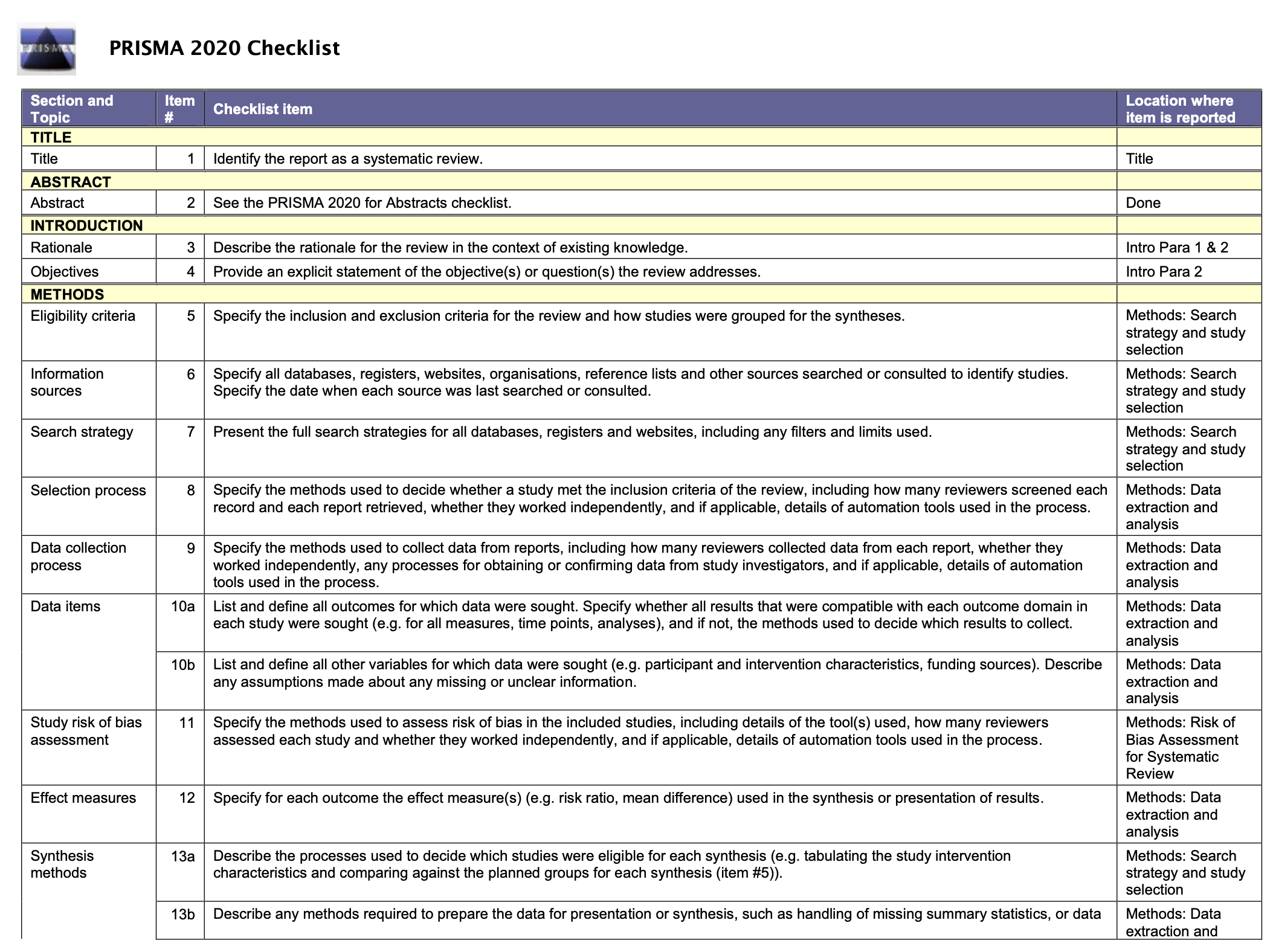

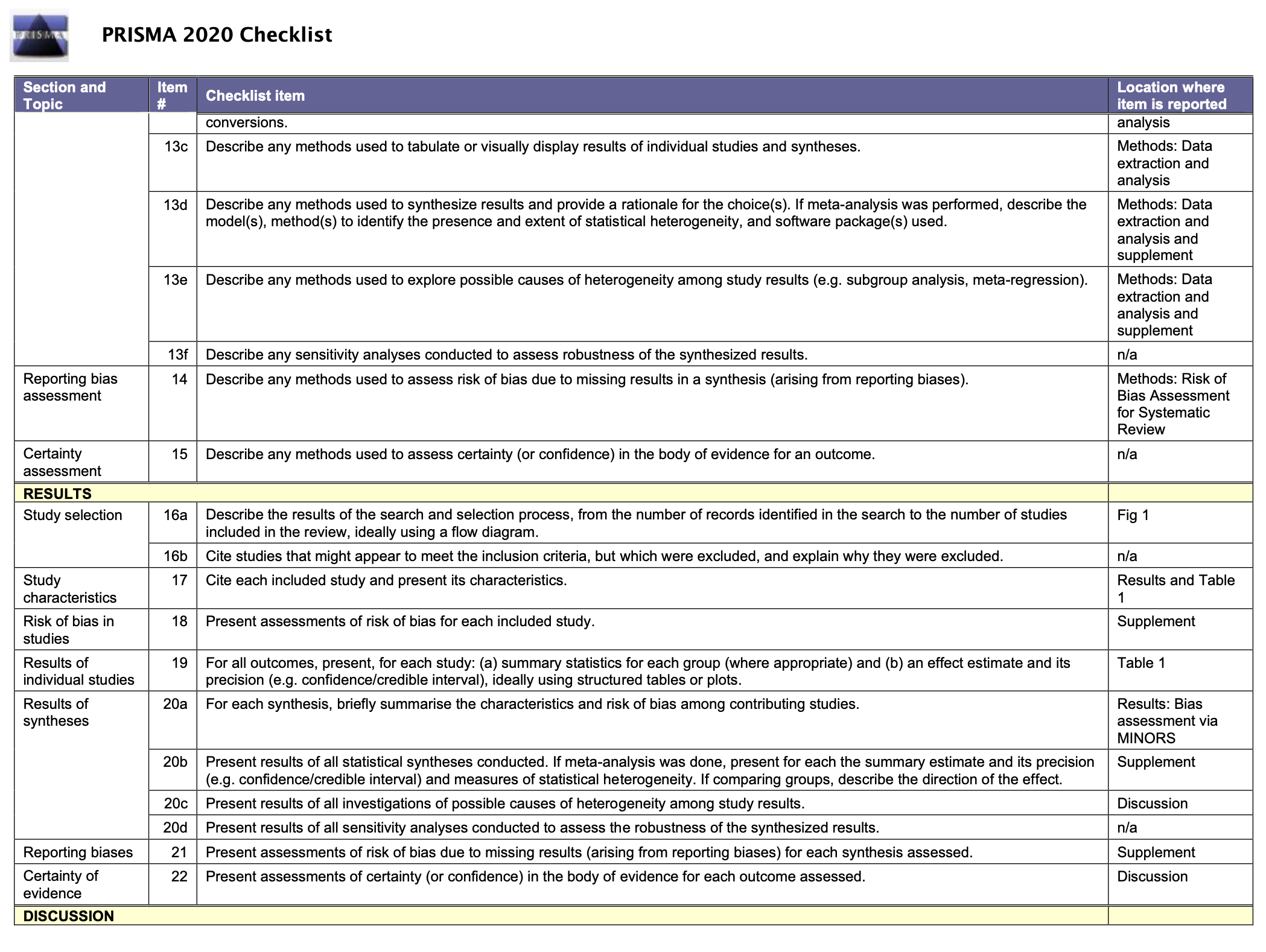

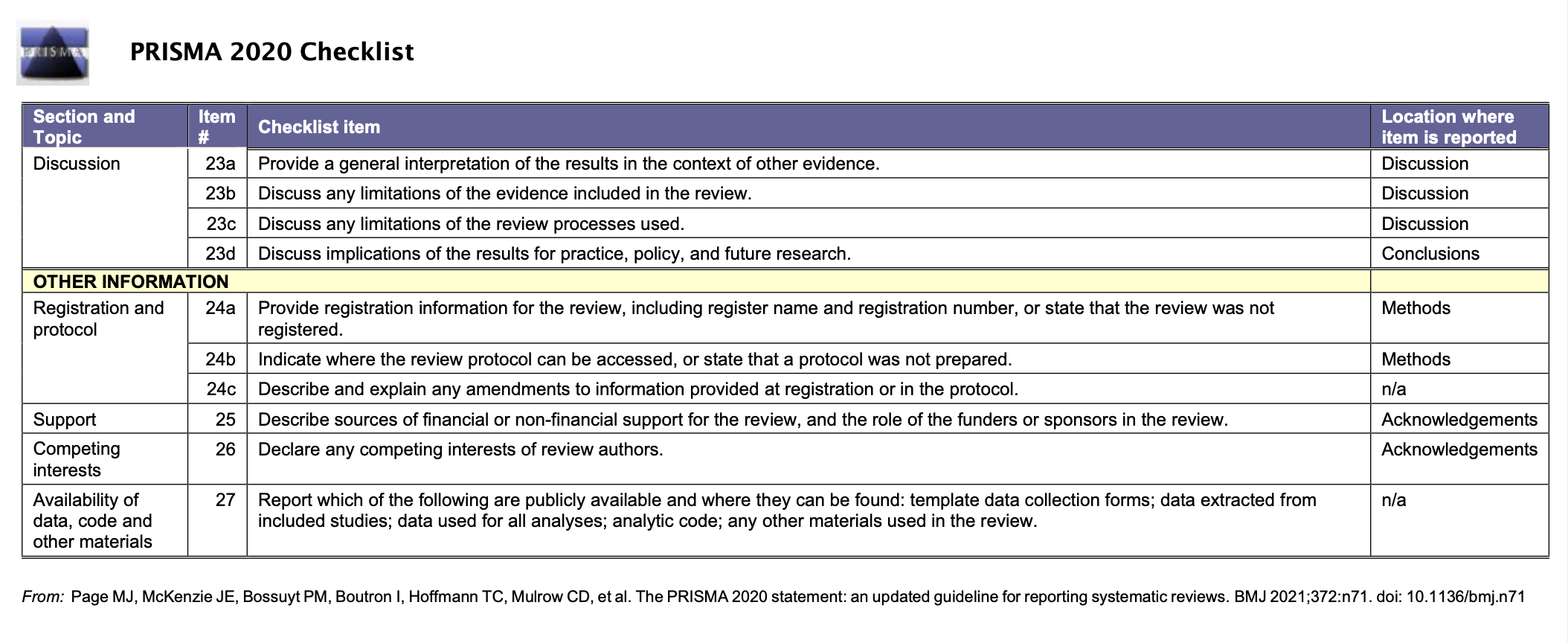

Supplement: Supplementary Material [file mmc1.docx]
